# Effect of *PEAR1, PTGS1* gene polymorphisms on the recurrence of aspirin-treated patients with ischemic stroke in the Han population of China: A 4-year follow-up study

**DOI:** 10.1097/MD.0000000000038031

**Published:** 2024-05-10

**Authors:** Linlin Zhang, Zhongru Meng, Hongxia Wang, Yang Miao

**Affiliations:** a Department of Pharmacology, the First People’s Hospital of Yancheng, Yancheng 224000, Jiangsu, China.

**Keywords:** aspirin, gene polymorphisms, ischemic stroke, *PEAR1*, *PTGS1*, recurrence

## Abstract

Platelet endothelial aggregation receptor 1 (*PEAR1*) and prostaglandin endoperoxide synthase 1 (*PTGS1*) polymorphisms can affect laboratory aspirin resistance. However, the impact of genetic polymorphisms on the recurrence of ischemic stroke (IS) patients treated with aspirin is not fully understood. This study aimed to examine the relationship between gene polymorphisms of *PEAR1* and *PTGS1* and IS recurrence in patients treated with aspirin. Peripheral blood samples were collected from 174 patients with nonrecurrent IS and 34 with recurrent IS after aspirin treatment. Follow-up was performed on all patients. *PEAR1 rs12041331* and *PTGS1 rs10306114* polymorphisms were determined using the PCR fluorescence probe method. And the correlations of them with the clinical characteristics were examined by multivariable logistic regression analysis. The distribution frequencies of *PEAR1 rs12041331* and *PTGS1 rs10306114* genotypes were in Hardy-Weinberg equilibrium, and there was no significant difference in the distribution of *PEAR1 rs12041331* polymorphism. Compared to the nonrecurrent group, the AA genotype of the *PTGS1* polymorphism was more frequent in the recurrent group (59.77% vs 35.29%, *P* = .003), and the A allele also showed a higher frequency than the G allele in the recurrent group (*P* = .001). Multivariable logistic regression analysis showed that smoking (OR = 5.228, 95% CI: 1.938–14.102, *P* = .001), coronary heart disease (OR = 4.754, 95% CI: 1.498–15.089, *P* = .008), and the polymorphism at *PTGS1(A>G) AA/AG* + *GG* (OR = 2.955, 95% CI: 1.320–6.616, *P* = .008) were independently associated with IS recurrence in Chinese patients. Our findings suggested that *PTGS rs10306114* polymorphisms should receive more attention in the use of aspirin in patients with IS.

## 1. Introduction

Ischemic stroke (IS) is a serious and prevalent disease that causes long-term disability and is a significant economic burden on individuals and society.^[1,2]^ In China, the incidence rate of cerebral infarction (IS) is the highest among stroke patients, accounting for 60% to 80%.^[3,4]^ Thirty percentage of patients with IS would experience secondary recurrence, and the risk of death or severe disability in patients with recurrent IS are 9.4 times higher than that in first-episode patients.^[5,6]^ Antiplatelet therapy can effectively prevent the occurrence and recurrence of IS.^[7]^ Aspirin is the most commonly used antiplatelet drug in clinical practice and is recommended as an initial treatment to prevent recurrent IS. However, in clinical practice, it has been found that some patients still experience cardiovascular and cerebrovascular events after long-term standardized use of aspirin after discharge, which is known as aspirin resistance (AR).^[8,9]^ After the occurrence of AR, platelets could still aggregate normally and the clotting time was not prolonged. One consequence was that, aspirin could not exert its expected preventive effect on thrombosis.^[10,11]^ Therefore, to improve the treatment of patients at risk of IS, it is important to further identify the risk factors of AR, enabling clinicians to choose more effective treatments for IS.

The mechanisms of AR are complicated, including noncompliance with aspirin therapy, cell-cell and drug-drug interactions, and coronary artery disease.^[12,13]^ In recent years, an increasing number of studies have shown that genetic polymorphisms in patients can affect AR.^[14–16]^ Studies have revealed that genetic polymorphisms of heme oxygenase 1 and cyclooxygenase-1 (COX-1) are associated with AR defined by light transmittance aggregation in Han Chinese patients.^[17]^ In addition, it was reported that platelet activity in Chinese patients with IS receiving aspirin therapy was closely related to genetic polymorphisms of *MDR1, TBXA2R*, and *PLA2G7*.^[18]^ The genes that affect AR include platelet endothelial aggregation receptor 1 (*PEAR1*) and prostaglandin endoperoxide synthase 1 (*PTGS1*: COX-1). As a platelet transmembrane protein, *PEAR1* involved in platelet aggregation and platelet-platelet contact, and was activated by platelet-to-platelet contact and agonist stimulation.^[19,20]^ Previous clinical studies have shown a strong link between genetic variations in *PEAR1* and platelet activity during aspirin treatment.^[21–23]^ Although genetic variant *PEAR1 rs12041331* was not associated with cardiovascular events in response to low-dose aspirin in a healthy elderly population, genotypes of *PEAR1* had impact on platelet reactivity in patients with recurrent IS treated with clopidogrel.^[24,25]^ In addition, *PTGS1* gene variations associated with bleeding and platelet dysfunction and *PTGS1* gene-smoking interaction might in part reflect the heterogeneity in the prognosis of stroke patients treated with aspirin.^[26]^ Nevertheless, the association between genetic variations in *PEAR1* and *PTGS1* and the recurrence of IS treated with aspirin has not been adequately evaluated.

In this study, we conducted a 1-year follow-up of newly diagnosed IS patients who received standard aspirin for secondary prevention after discharge. Focusing on the Chinese Han population, this study aimed to investigate the impact of *PEAR1 rs12041331* and *PTGS1 rs10306114* polymorphisms on the recurrence of IS in patients treated with aspirin to prevent or reduce the occurrence of IS.

## 2. Methods

### 2.1. General information of patients

This was a single-center, retrospective, case-control study of 243 patients with the first IS seen in consultation between July 2018 and June 2021 at the Department of Neurology of Yancheng First People’s Hospital. The study was approved by the ethics committee of Yancheng First People’s Hospital. The requirement for informed consent was waived by the committee because of the retrospective nature of the study. According to the actual situation, patients should take aspirin or a combination of aspirin and clopidogrel for the duration of hospitalization. After discharge, aspirin was administered in a standardized manner, and those with contraindications were replaced with clopidogrel.

The inclusion criteria were as follows: age ≥ 18 years; no requirement for sex; signs of acute IS, and diagnostic results of brain computed tomography (CT) or magnetic resonance imaging (MRI) met the diagnostic criteria for IS in the Chinese Guidelines for the Diagnosis and Treatment of Acute Ischemic Stroke 2018. The exclusion criteria were as follows: patients who were allergic to aspirin; patients with a history of peptic ulcer; cardiogenic cerebral infarction or hemorrhagic cerebral infarction; patients with blood system diseases; and taking drugs that affect platelet function.

A 1-year follow-up was performed for these patients, with 35 cases lost to follow-up and 208 cases ultimately included. The methods used for patient follow-up were as follows: 1 limb (with or without face) was weak or numb; one side of the face was numb or the corners of the mouth were crooked; difficulty speaking or understanding language; gaze sideways with both eyes; loss or blurring of vision in one or both eyes; dizziness with vomiting; rare severe headaches and vomiting in the past; and consciousness disorders or convulsions.

The procedures employed to identify recurrent IS events were CT findings and MRI findings. CT findings: within 24 hours of onset, CT scan could be negative, and later there will be slightly low-density areas with blurred edges, consistent with the occluded blood supply area (wedge-shaped or fan-shaped); within a week: lower density with significant occupancy; within 2 to 3 weeks: central necrosis, vascular proliferation, and reduced mass; blur effect, enhanced scan showing gyral enhancement; and weeks and months: cystic softening with brain atrophy. MRI findings: super acute phase (0–6 hours): DWI high signal, ADC low signal, T2 unchanged and showed equal signal; acute phase (6–72 hours): ADC showed low signal, DWI showed high signal and became brighter, and T2 showed high signal; subacute phase (3 to 10 days): the ADC value gradually increased, and around 10 days, the ADC could pseudonormalize and showed equal signals. DWI reached its brightest point at approximately 7 days and gradually darkened thereafter. The T2 signal gradually increased; chronic phase (>11 days): DWI had a low signal intensity and an elevated ADC value. T2 reached its peak after 7 to 30 days and gradually faded away thereafter.

All patients were recorded for sex, age, smoking history, drinking history, and other relevant information in detail, and were tested for *PEAR1* and *PTGS1* genotypes.

### 2.2. Grouping criteria

In view of whether the first-ever IS patients had a recurrence of stroke within 1 year after discharge, they were divided into a nonrecurrent IS group (174 cases) and a recurrent IS group (34 cases). Patients in the recurrent group were admitted to the hospital for the first IS from July 2018 to June 2021. Antiplatelet therapy (100 mg/1 time/d, Bayer Healthcare Co., Ltd., Sinopharm J20171021, 100 mg/tablet) was used for secondary prevention of stroke after discharge, and neurological deficits such as weakness or numbness of one limb (with or without face), slurred speech, or difficulty understanding language reappeared during the follow-up period. Head CT or MRI showed ischemic lesions in these patients.

### 2.3. Determination of *PEAR1* and *PTGS1* genotypes

Venous peripheral blood (2 mL of venous all patients). Subsequently, the blood samples were placed in EDTA anticoagulation tubes and tested using a PCR-fluorescent probe (Xi’an Tianlong Technology Co., Ltd.). An appropriate amount of universal sample diluent was then added to the test sample, mixed thoroughly, and set aside for later use. SNP-U7 positive and negative quality control samples did not require dilution.

The details are as follows: (1) an appropriate amount of universal sample diluent was added to the sample to be tested and well-mixed. (2) Taking 2 μL dilution, positive quality control and negative control samples were added to the reaction tube containing 3 kinds of PCR reaction solutions. (3) Centrifuged at 6000 rpm for 40 seconds in 37 °C warm bath for 3 minutes, vortexed again for 3 seconds, and then centrifuged at 6000 rpm for another 5 seconds. (4) Placed the reaction tube on the Fascan48E instrument (Xi’an Tianlong Technology Co., Ltd.) for sequencing. Amplification conditions: Predenaturation reaction at 94 °C for 3 minutes, denaturation reaction at the same temperature for 30 seconds, annealing reaction at 55 °C for 30 seconds, extension reaction at 72 °C for 30 seconds, repeating 35 cycles, and extension reaction at 72 °C for 5 minutes.

### 2.4. Statistical methods

Stata (version 14.0) software was used for statistical analysis, and the Shapiro–Wilk method was used for normality testing. The measurement data are expressed as mean ± standard deviation or median (interquartile range), and the counting data are expressed as frequency and percentage. Hardy-Weinberg equilibrium was tested for the *PEAR1 rs12041331* and *PTGS1 rs10306114* polymorphisms using the chi-square test. Categorical variables were reported as counts (percentages), and the chi-square test was used to compare groups. The *χ*^2^ test or Fisher exact test was used for comparison of counting data between the 2 groups, and the independent sample *t*-test or rank sum test was used for comparison between the measurement data groups. Multivariate analysis was performed using a logistic regression analysis (stepwise backward method). All statistical tests were 2-sided, and differences were considered statistically significant at *P* < .05.

## 3. Results

### 3.1. Characteristics of the patients

A total of 208 patients with IS treated with aspirin were included and divided into a nonrecurrent group (male, n = 121, 58.17%; female, n = 53, 25.48%) and a recurrent group (male, n = 22, 10.58%; female, n = 12, 5.77%). The demographic and clinical characteristics of the participants are summarized in Table 1. Multiple indicators showed differences between these 2 groups, including age, height, alcohol use, hypertension, diabetes, total cholesterol, triglycerides, LDL cholesterol, HDL cholesterol, homocysteine, platelets, and D-dimer. However, compared with the nonrecurrent group, the recurrent group showed significantly higher proportions of smokers and coronary heart disease patients.

**Table 1:** Characteristics of the patients (univariate analysis table).

	Nonrecurrent group (n = 174)	Recurrent group (n = 34)	*χ*^2^/*t*/*Z*	*P* value
Demographic data				
Sex				
Male, n (%)	121 (58.17%)	22 (10.58%)	0.309	.578
Female, n (%)	53 (25.48%)	12 (5.77%)		
Age (y)				
<50	19 (95.00%)	1 (5.00%)	−0.957	.338
50~60	30 (81.08%)	7 (18.92%)	−0.488	.625
60~70	52 (86.67%)	8 (13.33%)	−0.919	.358
70~80	54 (80.60%)	13 (19.40%)	−0.247	.805
80~	19 (79.17%)	5 (20.83%)	−1.624	.104
BMI (kg·m^-2^)				
<20	7 (87.50%)	1 (12.50%)	−1.537	.124
20~25	84 (84.00%)	16 (16.00%)	−0.485	.628
25~30	56 (86.15%)	9 (13.85%)	−1.178	.239
≥30	13 (76.47%)	4 (23.53%)	−2.943	.003
Blood lipid parameters				
LDL cholesterol (mmol·L^-1^)	2.23 (1.74,2.76)	2.39 (1.84,2.93)	−0.862	.39
HDL cholesterol (mmol·L^-1^)	1.04 (0.89,1.21)	0.98 (0.85,1.27)	0.316	.752
Total cholesterol (mmol·L^-1^)	4.00 (3.36,4.84)	3.95 (3.01,4.74)	0.635	.526
Total glyceride (mmol·L^-1^)	1.09 (1.44,2.12)	1.29 (1.02,2.09)	−0.271	.786
Homocysteine (µmol·L^-1^)	11.45 (9.2,14.5)	12.35 (10.08,15.65)	1.244	.214
Other blood parameters				
Platelet (10^9^·L^-1^)	173 (136.25,191.25)	179.5 (142.25,217.25)	1.259	.208
D-Dimer (mg·L^-1^)	0.74 ± 1.26	0.68 ± 0.80	−0.727	.467
Bad habits (n/%)				
Smoking	18 (10.34%)	4 (11.76%)	fisher	.764
Drinking	7 (4.02%)	3 (8.82%)	fisher	.088
Basic diseases (n/%)				
Hypertension	121 (69.54%)	22 (64.71%)	0.309	.578
Coronary heart disease	13 (7.47%)	3 (8.82%)	fisher	.73
Diabetes	45 (25.86%)	13 (38.24%)	2.165	.141

BMI = body mass index.

### 3.2. Genotypes

Genotype and allele frequencies of *PEAR1* and *PTGS1* are shown in Table 2. Among the 208 patients with IS, the incidence of *PEAR1 rs12041331* allele mutation was 38.22%, and the incidence of *PTGS1 rs10306114* allele variation was 25.72%. The distribution frequencies of each genotype met the Hardy-Weinberg equilibrium (*P* > .05), indicating that the sample was representative of the population.

**Table 2:** Hardy-Weinberg equilibrium test.

SNP (n = 208)	Genotypes	Real number (n)	Theoretical number (n)	*X*^2^ value	*P* value	Genotypes frequency (%)	Allele	Frequency (n)	Allele frequency (%)
*PEAR1 rs12041331*	*GG*	77	79			37.02	*G*	257	61.78
	*GA*	103	98	0.217	.897	49.52	*A*	159	38.22
	*AA*	28	30			13.46			
*PTGS1 rs10306114*	*AA*	116	113			55.77	*A*	307	73.80
	*AG*	75	80	0.489	.783	36.06	*G*	109	25.72
	*GG*	17	14			8.17			

Table 3 showed that there was a significant difference in the distribution of *PTGS1 rs10306114* polymorphism between nonrecurrent group and recurrent group (*P* = .000). In the nonrecurrent group, *AA* genotype accounted for 59.77%, *AG* genotype was 34.88%, and *GG* genotype made up 5.75%. While, *AA* accounted for 35.29% in the recurrence group, in which *AG* was 44.12%, and *GG* was 20.59%. However, nonrecurrent group and recurrent group showed no significant difference in the distribution of *PEAR1 rs12041331* polymorphism (*P* > .05). In the nonrecurrent group, the wild type *GG* accounted for 38.51%, the heterozygous mutant *GA* was 49.43%, and the homozygous mutant *AA* was 12.07%. The proportions of *GG, GA*, and *AA* in the recurrence group were 29.41%, 50.00%, and 20.59%, respectively.

**Table 3:** *PEAR1 rs12041331* and *PTGS1 rs10306114* genotype distribution and allele comparison.

			Nonrecurrent group (n = 174)	Recurrent group (n = 34)	*χ* ^2^	*P* value
*PEAR1 rs12041331*	Genotypes	*GG*	67 (38.51)	10 (29.41)	2.171	.338
		*GA*	86 (49.43)	17 (50.00)		
		*AA*	21 (12.07)	7 (20.59)		
	Allele	*G*	220 (63.22)	37 (67.70)	0.007	.933
		*A*	128 (36.78)	21 (36.21)		
*PTGS1 rs10306114*	Genotypes	*AA*	104 (59.77)*	12 (35.29)*	11.453	.003
		*AG*	60 (34.48)	15 (44.12)		
		*GG*	10 (5.75)†	7 (20.59)†		
	Allele	*A*	268 (77.01)	39 (57.35)	11.369	.001
		*G*	80 (22.99)	29 (42.65)		

* The 2 groups of wild-type AA/mutant (AG + GG) were compared, *χ*^2^ = 6.908, *P* = .009.

† The 2 groups were compared with GG/AA + AG, *χ*^2^ = 8.347, *P* = .004.

### 3.3. Association of the *PTGS1 rs10306114* polymorphism and other factors with recurrent IS by multivariable analysis

Taking the recurrence of first-episode IS patients within 1 year of discharge follow-up as the dependent variable and using 2 factors with significant differences in univariate analysis (smoking and coronary heart disease) and *PTGS1 rs10306114* mutation (*AG* *+* *GG*) as independent variables, we conducted a multivariate logistic regression analysis. As shown in Table 4, smoking (OR = 5.904), coronary heart disease (OR = 7.067), and *PTGS1 rs10306114 (A>G) AA/AG* + *GG* (OR = 2.528) were independently related factors for the recurrence in patients treated with aspirin.

**Table 4:** Multivariate logistic regression analysis of factors associated with recurrence in ischemic stroke.

Factor	OR	*Z* value	*P* value	95% CI
Smoking	5.228	3.270	.001	1.938–14.102
Coronary heart disease	4.754	2.650	.008	1.498–15.089
*PTGS1 rs12041331 (A>G*) *AA/AG + GG*	2.955	2.640	.008	1.320–6.616

## 4. Discussion

The impact of genetic polymorphisms on IS recurrence in patients treated with aspirin has not yet been fully elucidated. Focusing on the Chinese Han population, our research was devoted to clarify the relationship between the *PEAR1 rs12041331* and *PTGS1 rs10306114* polymorphisms and IS recurrence in patients treated with aspirin. Our results showed that polymorphisms at *PTGS1 rs10306114 (A>G) AA/AG* + *GG*, smoking, and coronary heart disease were independently associated with IS recurrence of IS treated with aspirin. To improve patient management and prognosis after a first ischemic stroke, the assessment of genetic polymorphisms in the prediction of IS recurrence in patients treated with aspirin warrants further investigation.

Aspirin can reduce thromboxane A2 synthesis by inhibiting the activity of COX-1 and exerting an anti-platelet aggregation effect, where COX-1 is encoded by the *PTGS1* gene.^[27–29]^ Clinical studies have reported that the *PTGS1 rs10306114* polymorphism is associated with AR in Chinese patients with cardio-cerebrovascular disease.^[30,31]^ There was an interaction between COX-1 and COX-2 variants, and this interaction was associated with AR in Chinese stroke patients.^[32]^ In this study, our results showed that the proportion of *PTGS1* mutations (*AG* + *GG*) in recurrent IS patients was significantly higher than that in nonrecurrent patients, and *PTGS1 rs10306114* mutations had a significant impact on IS recurrence. The probability of IS recurrence in patients with the *PTGS1 rs10306114* genotype was 2.955 times higher than that in wild-type patients.

*PEAR1* is highly expressed in platelets and endothelial cells, and phosphorylation of PEAR1 could cause irreversible aggregation reactions between platelets.^[33,34]^ It has been demonstrated that *PEAR1* polymorphisms could be a prognostic factor for hemostasis and cardiovascular diseases, and they also participate in multiple diseases, such as pulmonary thromboembolism and acute coronary syndrome.^[35–37]^ Studies have identified that *PEAR1* mutations have an impact on platelet function and antiplatelet drug efficacy.^[38]^ In addition, platelet reactivity is closely related to *PEAR1* polymorphisms in dual antiplatelet therapy in Chinese patients.^[39,40]^ Existing studies of 2007 patients have shown that *PEAR1 rs12041331* mutations were associated with AR via influencing platelet reactivity in Amish individuals.^[41,42]^ Regarding IS, *PEAR1* AA was shown to be an independent factor for short-term functional outcomes in patients with small-artery occlusion stroke treated with aspirin.^[43]^ However, no association was observed between platelet activity during aspirin therapy and *PEAR1 rs12566888/rs12041331* in a study of 283 ischemic stroke patients.^[18]^

Our study specifically focused on the Chinese Han population and found that *PEAR1 rs12041331* polymorphism had no effect on the recurrence of IS in aspirin-treated patients in the Chinese population. And demographic data, blood lipid parameters, other blood parameters, and basic diseases were the influence factors in our study. Previous study had used the univariable and multivariable Cox proportional hazard models, and 1-way ANOVA to carry out statistical analysis, and NYHA classification, Killip classification, and SYNTAX score were conducted in statistical analysis.^[41]^ It was reported that racial differences have a significant impact on gene polymorphisms, resulting in different responses to drug.^[44]^ Thus, we speculated that the reason for the differences in research conclusions might partly be the population, small sample size, insufficient consideration and differences in statistical methods during the research process. However, based on the real number and theoretical number of sample sizes, our data showed that the distribution frequencies of each genotype met the Hardy-Weinberg equilibrium (*P* > .05), which indicated that our sample size was still acceptable with a good justification.

Our results highlighted the impact of *PEAR1 rs12041331* and *PTGS1 rs10306114* on the recurrence of IS in Chinese patients treated with aspirin. We identified that smoking, coronary heart disease, and the polymorphism at *PTGS1 rs10306114 (A>G) AA/AG* + *GG* were independently associated with IS recurrence in Chinese patients. When AR occurred, platelets could still aggregate normally and the clotting time was not prolonged. Genetic polymorphisms of *PEAR1* and *PTGS1* induce AR by affecting COX-1, causing aspirin to fail to exert its expected preventive effect on blood clots. However, this research has several shortcomings. First, the small sample size may have impacted the conclusion. Thus, future studies need to expand the sample size to further confirm the conclusions of this study. Second, it is well known that there are genetic polymorphisms among different ethnic populations, and Chinese Han patients were our research subjects. Therefore, our conclusions cannot be generalized to other ethnic populations. Third, besides *PEAR1 rs12041331* and *PTGS1 rs10306114*, other gene polymorphisms might also be associated with IS recurrence in patients treated with aspirin. To make the conclusions of this study more convincing, more influencing factors should be incorporated in future research.

In addition to aspirin, pharmacogenomics has already uncovered many links between genetic variation and the response to other anticoagulant drugs, such as *SLCO1B1, ABCB1, CYP3A4, CYP3A5, CYP2C19, PTGS1, PTGS2, ADRB1, ADCY9, CYP2C19, PON1, CES1, GPIIIa, CYP2D6, CKORC1*, and *CYP2C9*.^[45]^ *PEAR1* polymorphisms have been reported to affect the risk of adverse effects and the response efficacy of clopidogrel and warfarin. The present study revealed that *PTGS1 rs10306114* polymorphism is closely related to IS recurrence in patients treated with aspirin. The AA genotype of the *PTGS1 rs10306114* polymorphism was more frequent and the A allele also showed a higher frequency than the G allele in patients with recurrent IS treated with aspirin. Smoking, coronary heart disease, and *PTGS1 rs10306114 (A>G) AA/AG* + *GG* were independently associated with aspirin recurrence in patients with IS in the Chinese population. Thus, genetic variation should receive more attention in the use of anticoagulant drugs in patients with IS, besides *PTGS rs10306114*.

## Author contributions

**Conceptualization:** Linlin Zhang, Yang Miao.

**Formal analysis:** Zhongru Meng.

**Methodology:** Zhongru Meng.

**Project administration:** Zhongru Meng.

**Writing – original draft:** Linlin Zhang.

**Writing – review & editing:** Hongxia Wang, Yang Miao.
